# Editorial: Humanoid Robots for Real-World Applications

**DOI:** 10.3389/frobt.2022.938775

**Published:** 2022-06-07

**Authors:** Fumio Kanehiro, Wael Suleiman, Robert Griffin

**Affiliations:** ^1^ CNRS-AIST Joint Robotics Laboratory (JRL), IRL3218, National Institute of Advanced Industrial Science and Technology (AIST), Tsukuba, Japan; ^2^ Electrical and Computer Engineering Department, Université de Sherbrooke, Sherbrooke, QC, Canada; ^3^ Florida Institute for Human and Machine Cognition, Pensacola, FL, United States

**Keywords:** humanoid robots, industrial robots, collaborative robots, avatar robot, whole-body control, posture generation

Since Honda introduced the P2 in 1996, numerous humanoid robots have been developed around the world, and research and development of various fundamental technologies, including bipedal walking, have been conducted. At the same time, attempts have been made to apply humanoid robots to various applications such as plant maintenance, telemedicine, vehicle operation, home security, construction, aircraft manufacturing, disaster response, evaluation of assistive devices, and entertainment.

Humanoid robots have an anthropomorphic body, and their major advantage is that they can move within an environment designed for humans and can use tools and vehicles designed for humans as they are. It is hoped that these advantages can be used to help people focus on more creative activities by replacing activities in harsh environments, hazardous tasks, and low added-value tasks that people are forced to perform because existing fixed, wheeled, or crawler-type robots are unable to deal with them.

In addition, since the fact that something shaped like a human moves like a human has an effect of attracting people, it can be expected to entertain and heal people by interacting with them. It is easier for humans to understand their “intention” through body language. This is related to the avatar application introduced later.

Despite these expectations, even today, more than 25 years after the announcement of P2, there is still no humanoid robot that has been put to practical use other than R&D and communication applications. This is because there is no necessity to use humanoid robots in a structured environment like a conventional factory, where existing robots can be easily applied, and the technology is too immature to use humanoid robots in an environment that is so unstructured that existing robots cannot deal with.

This Research Topic introduces two efforts to improve the basic capabilities of humanoid robots and one effort to apply humanoid robots to remote services, with the aim of practical applications of humanoid robots.

Until now, almost all humanoid robots have used a method in which joints are accurately position-controlled and position commands are updated using joint velocities calculated by inverse kinematics. Recently, methods that updates the position commands by calculating joint accelerations using inverse dynamics calculations, and methods that control the joint torques are being used. Ramuzat et al. implemented these three approaches on the same hardware platform and clarified the advantages and disadvantages of each approach. The method combining position control and inverse kinematics was found to be the least computationally intensive, while the method using torque control was confirmed to have advantages in terms of smoothness of trajectory tracking, energy consumption, and passivity. Recent improvements in computer performance have made it possible to perform inverse dynamics-based torque control at 1 kHz, and there is a possibility that torque control will become the mainstream of joint control in the future.

Multi-contact technology is essential to enable humanoid robots to move in unstructured environments where it is difficult for existing robots to operate. By actively bringing various parts of the body into contact with the environment, humanoid robots can move in confined spaces that are inaccessible to wheeled robots with large footprints. One of the basic functions in multi-contact motion generation is the Posture Generator. This is the problem of calculating joint angles that can realize a given set of contacts without colliding with the environment or the robot itself, and because it is a process that is called frequently in multi-contact motion generation, it must be computationally fast. In the past, collision avoidance was often incorporated into the inverse kinematics solver as an inequality constraint. However, when there are many obstacles in proximity, such as narrow passages, the number of constraints increases, and the computation speed slows down. Rossini et al. tackled the latter problem by proposing a method to generate a collision free posture using an adaptive random velocity vector generator and showed that it is effective especially in narrow environments.

Due in part to the influence of COVID-19, the use of avatar robots to provide remote services has attracted much attention in recent years. Baba et al. compared the performance and perceived workload of face-to-face service delivery and service delivery via avatars in a public space. They found no significant difference in performance, but interestingly found that the perceived workload was smaller when the service was provided via an avatar robot.

Further research and development are needed to enable humanoid robots to autonomously perform tasks that are currently difficult for other robots, and it is expected that it will take more time to achieve this goal. To promote the industrialization of humanoid robots as early as possible, it is thought that their deployment as avatar robots would be an effective way. Using the robot as an avatar robot will enable humans to perform tasks that are harsh or dangerous while being in a safe and comfortable environment. Moreover, it will also enable humans to compensate for the robot’s insufficient abilities, such as advanced situational awareness and higher-level decision making, through remote control. By starting industrialization of humanoid robots in this manner and utilizing them in the real fields every day, a virtuous cycle can be expected to emerge whereby costs are reduced while reliability and autonomy are improved.

